# A drug-repurposing screen of FDA- and EMA-approved drugs identifies two NF-κB inhibitors active against eumycetoma

**DOI:** 10.1093/jac/dkaf369

**Published:** 2025-09-29

**Authors:** Jingyi Ma, Bjorn R van Doodewaerd, Bernhard Biersack, Annelies Verbon, Paul P Geurink, Wendy W J van de Sande

**Affiliations:** Department of Medical Microbiology and Infectious Diseases, Erasmus MC, University Medical Center Rotterdam, Rotterdam, The Netherlands; Department of Cell and Chemical Biology, Leiden University Medical Center, Leiden, The Netherlands; Organic Chemistry Laboratory, University Bayreuth, Bayreuth, Germany; Department of Medical Microbiology and Infectious Diseases, Erasmus MC, University Medical Center Rotterdam, Rotterdam, The Netherlands; Department of Cell and Chemical Biology, Leiden University Medical Center, Leiden, The Netherlands; Department of Medical Microbiology and Infectious Diseases, Erasmus MC, University Medical Center Rotterdam, Rotterdam, The Netherlands

## Abstract

**Background:**

Mycetoma caused by *Madurella mycetomatis* (eumycetoma) is a neglected tropical disease that forms tumorous lesions in the subcutaneous tissue. The current standard treatment for eumycetoma consists of antifungal treatment combined with surgery, with limited success rates. Due to lack of investment, there are currently no large drug discovery programmes for mycetoma. Repurposing screening can therefore offer an effective and economical way to identify drugs that can be effective in eumycetoma treatment. Therefore, in this study we determined the *in vitro* activity and *in vivo* efficacy of 5631 compounds present in the Oncode Drug Repurposing library.

**Methods:**

In total, 5631 drugs from the Oncode Drug Repurposing library were screened for *in vitro* activity against *M. mycetomatis* using a CLSI-based *in vitro* susceptibility assay and CellTiter-Glo as a viability dye. Compounds that inhibited the metabolic activity were tested for *in vivo* activity in *M. mycetomatis*-infected *Galleria mellonella* larvae.

**Results:**

Twenty-eight compounds out of the 5631 drugs were able to inhibit the metabolic activity of *M. mycetomatis* at 2 µM. Seventeen of the 28 compounds were azoles and 2 were toxic to *G. mellonella* and therefore not screened further. Two from the remaining 9 compounds, bay117085 (log-rank, *P* = 0.0494) and IMD-0354 (log-rank, *P* = 0.0043), prolonged the survival of *M. mycetomatis*-infected larvae. Both compounds were designed as NF-κB inhibitors.

**Conclusions:**

NF-κB inhibitors bay117085 and IMD-0354 were able to prolong the survival of *M. mycetomatis*-infected larvae.

## Introduction

Mycetoma is a neglected tropical disease that is characterized by subcutaneous tumorous lesions, most often in the feet.^1^ It is caused by either bacteria (actinomycetoma) or fungi (eumycetoma). A characteristic of mycetoma is that the causative agents will organize themselves in protective structures called grains.^2^ Actinomycetoma is most commonly caused by the actinomycetes *Nocardia brasiliensis*, *Actinomadura madurae* and *Streptomyces somaliensis* and form white to yellow grains.^3^ In 85% of the reported cases, eumycetoma is caused by the fungus *Madurella mycetomatis*, which forms black grains.^4^ Within these grains the fungus is embedded in a cement material.^2^ Due to the evolutionary differences in causative agents, treatment for actinomycetoma and eumycetoma also differ. Actinomycetoma is treated by a combination of trimethoprim/sulfamethoxazole and amikacin, which has a cure rate of >90%.^3^ Eumycetoma is treated by a combination of treatment with itraconazole and surgery. In a clinical trial setting, the cure rate of this regimen is 80%; however, in real life, cure rates are much lower, with corrected cure rates reported between 48% and 68%.^2,5^ This difference is most likely due to differences in compliance as mycetoma needs to be treated for more than a year, and in most endemic regions, mycetoma patients need to pay for the drugs themselves. Therefore, there is an urgent need to find an affordable drug that can result in shorter treatment regimens, and which will make the surgery redundant.

Developing novel drugs for a disease is a lengthy and expensive process and includes many steps in which a potential drug can fail. For neglected tropical diseases such as mycetoma, return of investment is limited and there is currently no specific drug discovery programme for mycetoma by pharmaceutical companies. Only fosravuconazole was clinically evaluated as a potential new drug for mycetoma, but with a similar outcome to itraconazole.^6–8^ Furthermore, surgery still needed to be performed. To identify more compounds, the open-source drug discovery programme for mycetoma (MycetOS) was established in 2018.^9^ In this programme, several compound libraries from the Medicines for Malaria Venture (MMV) were screened to identify compounds with activity against eumycetoma.^8,10,11^ However, the majority of the compounds in these boxes were selected based on their assumed activity against various bacteria, parasites and fungi. Moreover, most of these compounds were in the development stage and not approved by the FDA or the EMA for any disease, which means that for most of these compounds not even Phase I studies had been performed.

So far, to the best of our knowledge, no one has screened the activity of FDA- or EMA-approved drugs against *M. mycetomatis* using an *in vitro* screening approach. They have only been screened *in silico* against the CYP51 enzyme of *M. mycetomatis*.^12^ In 2017, Corsello *et al*. developed the Drug Repurposing Hub, in which a compound library was created with 4707 compounds, of which 1988 were approved for clinical use by the FDA and/or EMA, and 1348 were under clinical investigation for several human diseases.^13^ Since then, the Broad Institute collection has further expanded this library and it currently consists of 7934 compounds. The Dutch Oncode Institute created a library based on this library and made it available to Dutch researchers.^14^ This Oncode library consists of 5631 compounds (Table S1, available as Supplementary data at *JAC* Online) and can be obtained via a request to the Oncode institute via their website.^15^ Here, we aimed to discover new compounds with activity against *M. mycetomatis* by screening the Oncode library. We first determined if any of the 5631 compounds in this library were able to inhibit the growth of *M. mycetomatis in vitro*. For the most promising compounds, the *in vivo* efficacy was also determined in a *M. mycetomatis*-infected *Galleria mellonella* model. In this invertebrate model the characteristic *M. mycetomatis* black grains with cement material are formed.^16^

## Materials and methods

### Fungal strains and Galleria mellonella larvae

*M. mycetomatis* isolate MM55 was used to screen the Oncode library. This strain was kindly provided by Prof. A. Fahal of the Mycetoma Research Centre in 1999. The most promising hit compounds were also screened against *M. mycetomatis* isolates P1 and I1, which originated from Mali and India, respectively.^17^ The three *M. mycetomatis* isolates were identified by ITS sequencing and maintained at −80°C at Erasmus MC. Prior to *in vitro* screening, isolates were cultured on Sabouraud agar plates (BD, Erembodegem, Belgium) at 37°C.

For *in vivo* experiments, sixth-week instar *G. mellonella* larvae were purchased from Blue-Lagoon, Maassluis, The Netherlands. The larvae were raised and kept in the dark at room temperature. Larvae without any discoloration and weighing around 500 mg were selected for experiments.

### Oncode library

The Oncode Drug Repurposing library was based on the drug repurposing library from the Drug Repurposing Hub of the Broad Institute and recreated by the Oncode institute.^13^ The library contained 5631 drugs.^18^ A copy of this library was plated out on 384-well plates with lids (cat no. 655180; Greiner Bio-One, Alphen aan den Rijn, The Netherlands) by the Leiden University Medical Center. Each well of the 384-well plates contained 0.6 µL of a 200 µM stock concentration of compound, resulting in a final concentration of 2 µM when the fungus was added. From the previous libraries we screened, we noted that there are several compounds able to inhibit *M. mycetomatis* at this concentration *in vitro*. Furthermore, this concentration could also be reached in the *G. mellonella* larvae.^8,11^ The prepared 384-well plates were transferred on dry ice to the Erasmus MC and stored at −80°C until they were screened. We created a second library from the original library, containing only those 29 compounds that were active in the first screen. These 29 compounds were plated out in 0.6 µL of 200, 100, 50, 25, 12.5, 6.25 and 3.1 µM concentrations to be able to determine the concentrations in which 50% of the fungal hyphae were inhibited in growth (IC_50_) and at which concentration the fungi were completely inhibited in growth (MIC). The list of compounds present in this library is provided in Table S1.

### Compounds

For *in vivo* efficacy testing, the compounds active in the first screen were obtained from pharmacological companies. IKK2 inhibitor V [IMD-0354 (cat. no. 978-62-1)], broxyquinoline (521-74-4), clioquinol (130-26-7), bay117085 (196309-76-9) cgp60474 (164658-13-3), broxaldine (3684-46-6), nsc319726 (71555-25-4) and thimerosal (54-64-8) were all obtained from Bio-Connect B.V., Huissen, The Netherlands. Phenylmercuric acetate (P27127) was obtained from Sigma–Aldrich, Japan. Tyrphostin 9 (tyrphostin A9, malonoben) was obtained from Sigma–Aldrich, Germany.

### *In vitro* susceptibility testing

To determine the *in vitro* susceptibility of *M. mycetomatis* to the compounds, a hyphal inoculum was prepared as described elsewhere.^19^ This *M. mycetomatis* hyphal suspension (60 µL) was added to each of the wells in the 384-well plate, leading to a final testing concentration of 2 µM per compound. Each plate also contained a growth control (60 µL of *M. mycetomatis* hyphal suspension, plus 0.6 µL of DMSO) and a negative control (60 µL of culture medium, plus 0.6 µL of DMSO). Plates were then incubated for 4 days at 37°C, after which 30 µL of CellTiter-Glo 3D Cell Viability reagent was added to every well.^19^ After 25 min of incubation at room temperature, the luminescence was measured with a CytoFluor Series 4000 luminometer (PerSeptive Biosystems, Framingham, MA, USA). To determine the metabolic activity, the following formula was used:


$$Percentage\;metabolic\;activity=\left(\frac{Absorbance\;test_{luminensence}-Absorbance\;NC_{luminensence}}{\;Absorbance\;GC_{luminensence}-Absorbance\;NC_{luminensence}}\right)\times 100$$

where, GC is growth control and NC is negative control. The luminescence measured for the GC is considered as 100% metabolic activity. The luminescence for the NC is considered as 0% metabolic activity. When a compound resulted in a metabolic activity of 20% or below, that compound was considered to inhibit the fungal growth.

### Toxicity testing in *G. mellonella* larvae

Compounds that were able to inhibit *M. mycetomatis* growth at 2 µM or below were tested for toxicity. To determine the toxicity, 15 healthy uninfected larvae were injected with 20 µL of 20 µM compound as previously described.^16^ For this, 20 µL of 20 µM IKK2 inhibitor V (IMD-0354), broxyquinoline, clioquinol, bay117085, cgp60474, broxaldine, nsc319726, thimerosal, phenylmercuric acetate and tyrphostin 9 were injected into the last left proleg of healthy uninfected larvae via an insulin 29G U-100 needle (BD Diagnostics, Sparks, MD, USA). As a control, a separate group of larvae were injected with 20 µL of PBS. Larvae were housed in Petri dishes with Whatman paper at 37°C in the dark. Survival was monitored for 10 days. A compound was considered toxic if more than 20% of the larvae died in the 10 day observation period. When compounds were toxic at 20 µM, toxicity experiments were repeated with 10, 5, 1 and 0.5 µM solutions.

### Infection and treatment in *G. mellonella* larvae

To determine the therapeutic efficacy in *M. mycetomatis*-infected larvae, *G. mellonella* larvae were infected with *M. mycetomatis* MM55 via the last proleg, and treatment was given for 3 days, as in our previously published protocol.^16^ In short, a fungal inoculum was prepared by culturing *M. mycetomatis* in a 500 mL RPMI1640 working medium for 2 weeks at 37°C and collecting the fungal mycelium via vacuum filtration over a 0.22 µm filter (Nalgene, Abcoude, The Netherlands). The wet weight was determined and 10 mL of PBS per gram of mycelia was added. The suspension was then sonicated for 2 min to achieve a particle size of 10 µm. The fungal suspension (4 mg/40 µL) was injected into the last left pro-leg of a healthy *G. mellonella* larva with an insulin 29G U-100 needle to reach a final inoculum of 4 mg/larva. Larvae were placed in Petri dishes containing Whatman paper in a 37°C incubator.

After 4 h, 20 µL of 20 µM compound [IKK2 inhibitor V (IMD-0354), broxyquinoline, clioquinol, bay117085, cgp60474 and broxaldine] or 20 µL of 10 µM compound (nsc319726, thimerosal and tyrphostin 9) was injected. A control group was also included in which *M. mycetomatis*-infected larvae were treated with PBS or with 1 mg/kg amphotericin B (Fungizone, Bristol Myers Squibb, Utrecht, The Netherlands). PBS will not influence the natural infection, and 1 mg/kg amphotericin B will result in prolonged survival.^16^ The treatment was repeated at 28 h post infection and 52 h post infection via different prolegs. The infection was monitored for 10 days.

### Statistical analysis

To compare the survival rates between PBS treatment control and the different experimental groups a log-rank test was performed. All data were processed and performed in GraphPad Prism 10. A *P* value of <0.05 was considered significant and shown as *, and a *P* value of <0.01 was considered significant and shown as **.

## Results

### Twenty-nine of 5631 compounds were able to inhibit the growth of *M. mycetomatis* at a concentration of 2 µM

**Figure 1.**
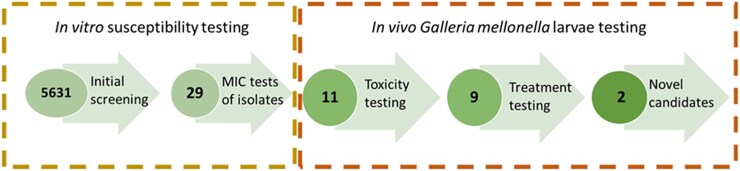
Flow diagram representing the *in vitro* and *in vivo* screening process used to identify novel drug candidates for eumycetoma caused by *M. mycetomatis*. Initially, 5631 compounds in the Oncode library were tested for their ability to inhibit the growth of *M. mycetomatis* at 2 µM. From this initial screening, 29 compounds of 5631 compounds were able to inhibit the growth of *M. mycetomatis*. Of these 29 compounds, those belonging to the azole class were excluded from further analysis. The 11 novel compounds were selected for *in vivo* toxicity testing. Nine compounds appeared to be non-toxic and were screened for *in vivo* efficacy in *M. mycetomatis*-infected *G. mellonella* larvae. Finally, two of these compounds appeared to prolong the survival of *M. mycetomatis*-infected larvae.

From the 5631 compounds present in the Oncode library, 29 compounds were able to inhibit the metabolic activity of *M. mycetomatis* strain MM55 at a concentration of 2 µM (Figure 1, Table 1). From these 29 compounds, 17 were azoles, namely voriconazole, isoconazole, flutrimazole, efinaconazole, enilconazole, isavuconazole, ravuconazole, itraconazole, miconazole, oxiconazole, clotrimazole, cloconazole, ketoconazole, tioconazole, posaconazole, lanoconazole and luliconazole. The remaining 12 compounds were ncs319726, broxyquinoline, bay117085, clioquinol, IMD-0354 (IKK2 inhibitor V), bleomycin, thimerosal, cgp60474, tyrphostin 9, broxaldine, phenylmercuric acetate and alexidine dihydrochloride. All data of the 5631 compounds are shown in Table S1.

**Table 1.** Average metabolic activity, IC_50_ and MIC of the 29 compounds from the Oncode repurposing library with inhibitory activity at 2 µM

Compound name	Class	Application	Mode of action	Average metabolic activity at 2 µM (%)	SD	IC_50_ (µM)			MIC (µM)		
						strain mm55	strain P1	strain I1	strain mm55	strain P1	strain I1
nsc319726	Thiosemicarbazone	Anticancer agent (human)	Targets P53 pathway	4.1	2.8	1.61	0.74	1.04	2.00	2.00	2.00
Voriconazole	Triazole	Antifungal agent (human)	Targets CYP51	1.1	1.5	0.63	0.33	0.20	1.00	0.50	0.25
Isoconazole nitrate (Travogen)	Triazole	Antifungal agent (human)	Targets CYP51	1.2	1.7	1.42	0.06	0.36	2.00	0.50	0.50
Flutrimazole	Imidazole	Antifungal agent of topical fungal infections (human)	Targets CYP51	0.0	0.0	0.92	0.37	0.42	2.00	0.50	0.50
Broxyquinoline	8-Hydroxyquinolines	Antiprotozoal agent	Not known	3.3	3.3	1.60	0.56	1.29	2.00	2.00	2.00
Efinaconazole	Imidazole	Antifungal agent of topical fungal infections (human)	Targets CYP51	0.0	0.0	0.04	<0.03	0.05	0.06	0.03	0.06
Enilconazole sulphate	Ketoconazole derivative	Agriculture; veterinary medicine	Targets CYP51	1.3	1.8	1.25	0.56	0.42	2.00	1.00	0.50
Isavuconazole	Triazole	Antifungal agent	Targets CYP51	2.1	2.1	0.43	<0.03	0.05	1.00	0.03	0.06
bay117085	NF-kB	Immunomodulators	Targets P65 nuclear translocation	4.0	3.1	1.51	0.85	0.72	2.00	2.00	1.00
Ravuconazole	Triazole	Antifungal agent	Targets CYP51	1.3	0.4	0.17	<0.03	<0.03	0.25	0.06	0.03
Itraconazole hydrochloride	Triazole	Antifungal agent	Targets CYP51	6.1	1.8	1.58	0.84	0.78	2.00	2.00	1.00
Miconazole nitrate	Imidazole	Antifungal agent of topical fungal infections (humans and cats)	Targets CYP51	1.0	1.4	0.64	0.26	0.05	1.00	0.50	0.06
Clioquinol	8-Hydroxyquinolines	Zinc and copper chelator	Not known	0.0	0.0	1.20	0.38	1.70	2.00	0.50	2.00
Oxiconazole nitrate	Imidazole	Antifungal agent of topical fungal infections	Targets CYP51	0.4	0.6	0.22	<0.03	<0.03	0.50	0.06	0.03
IKK2 inhibitor V (IMD-0354)	Kinase inhibitor	Anticancer agent (human)	NF-κB inhibitor	2.3	0.4	1.76	0.17	1.68	2.00	0.25	2.00
Clotrimazole	Imidazole	Antifungal agent of topical fungal infections	Targets CYP51	4.0	1.5	0.86	0.49	0.36	1.00	1.00	0.50
Cloconazole HCl	Vinylimidazole	Antifungal agent of topical fungal infections	Targets CYP51	1.7	2.2	0.85	0.74	0.41	1.00	1.00	0.50
Bleomycin	Glycopeptide antibiotics	Antineoplastic agent	Targets DNA synthesis	1.6	2.3	0.21	<0.03	0.20	0.50	0.03	0.25
Thimerosal	Alkylmercury	Germicide	Not well known	1.4	1.9	0.08	<0.03	0.04	0.25	0.03	0.06
Ketoconazole	Imidazole	Antifungal agent of topical fungal infections	Targets CYP51	0.9	1.3	0.66	0.17	0.05	1.00	0.25	0.06
Tioconazole	Imidazole	Antifungal agent of topical fungal infections	Targets CYP51	3.2	0.2	1.24	0.39	0.33	2.00	0.50	0.50
cgp60474	Kinase inhibitor	Anti-endotoxaemia agent	NF-κB inhibitor	7.4	2.4	1.55	0.64	0.81	2.00	1.00	1.00
Broxaldine	Tetracycline class of antibiotics	Antiprotozoal agent	Protein ligand-linker conjugate	4.8	2.0	1.25	0.63	1.31	2.00	1.00	2.00
Posaconazole	Triazole	Antifungal agent	Targets CYP51	1.9	1.6	0.13	<0.03	<0.03	0.25	0.03	0.03
Phenylmercuric acetate	Organomercury compound	Fungicide and slimicide	Not well known	2.0	2.9	1.12	<0.03	0.45	2.00	0.03	0.50
Lanoconazole	Imidazole	Antifungal agent of topical fungal infections	Targets CYP51	3.0	1.3	0.04	<0.03	<0.03	0.06	0.03	0.03
Alexidine dihydrochloride	Biguanide	Antimicrobial agent	Targets a mitochondrial tyrosine phosphatase, PTPMT1	4.4	2.6	1.32	1.78	0.80	2.00	>2	>2
Luliconazole	Imidazole	Antifungal agent of topical fungal infections	Targets CYP51	1.0	0.4	<0.03	<0.03	<0.03	0.03	0.03	0.03
Tyrphostin 9	Kinase inhibitor	Anti-inflammatory agent	Inhibition of tyrosine phosphorylation in signal transduction pathways	1.5	0.3	1.36	1.22	0.78	2.00	2.00	1.00

For each of these 29 active compounds, the IC_50_ and MIC were determined for *M. mycetomatis* strains MM55, P1 and I1 (Table 1 and Table S1). As can be seen in Table 1, the three most potent compounds were posaconazole, luliconazole and lanoconazole, with a mean MIC of <0.03 µM. The least active compound was alexidine dihydrochloride, with a mean MIC above 2 µM, and therefore, this compound was not tested further.

### Nine of 11 selected compounds were not toxic for *G. mellonella* larvae

**Figure 2.**
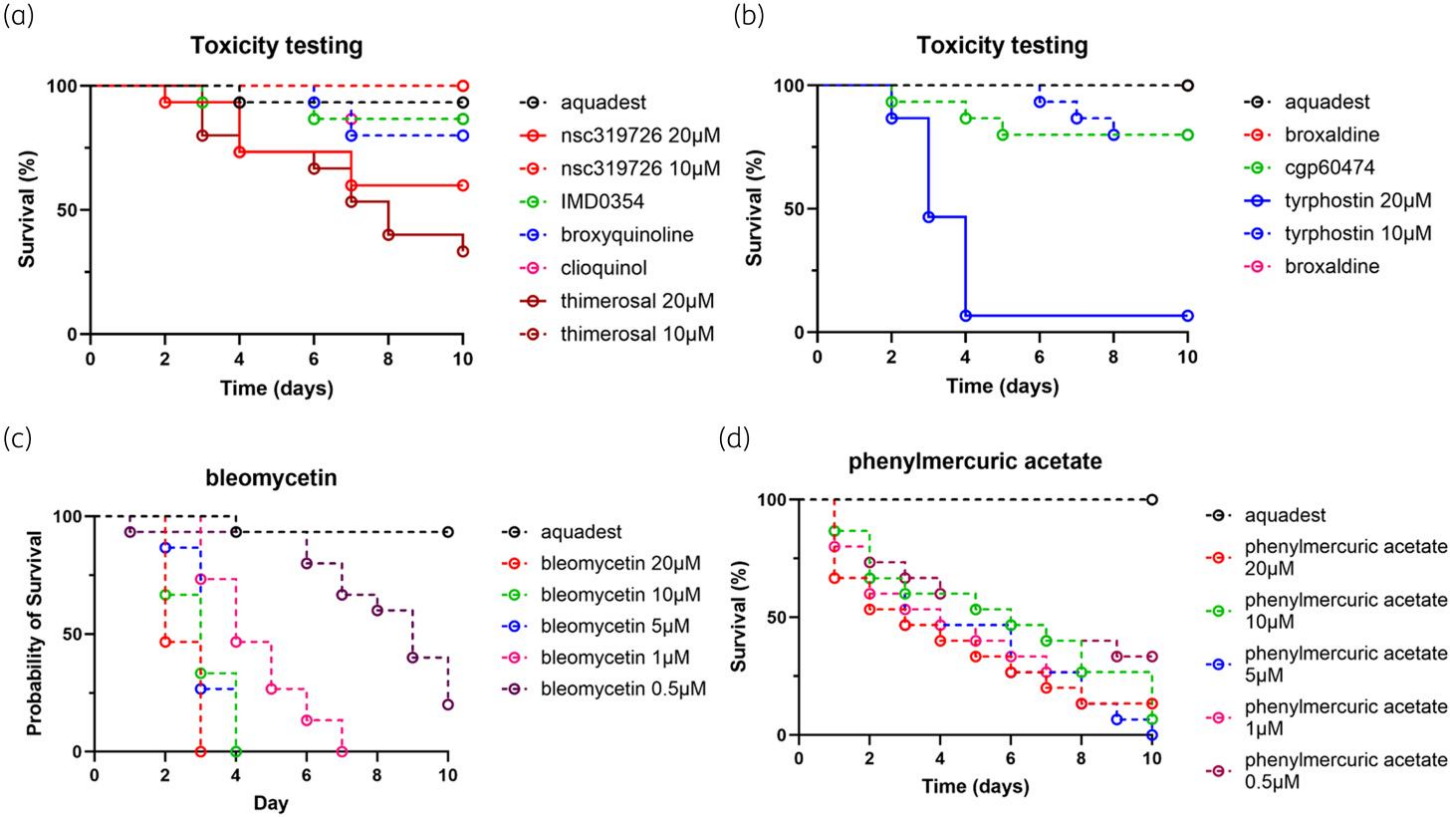
Toxicity testing of the 11 selected compounds. To determine the toxicity of the selected compounds, 20 µM compound was injected into *G. mellonella* larvae. When compounds appeared to be toxic, the concentrations were lowered to 10, 5, 1 and 0.5 µM and tested again. (a) Toxicity testing for IMD-0354 (green dashed line), broxyquinoline (blue dashed line) and clioquinol (purple dashed line). ncs319726 (red line) and thimerosal (dark red) were tested at both 20 µM (solid line) and 10 µM (dashed line). (b) Toxicity testing for tyrphostin 9 (blue solid line), bay117085 (red dashed line), cgp60474 (green dashed line) and broxaldine (purple dashed line) at 20 µM. Tyrphostin 9 was also tested at 10 µM (blue dashed line). (c) Toxicity testing for bleomycin at 20 µM (red dashed line), 10 µM (green dashed line), 5 µM (blue dashed line), 1 µM (purple dashed line) and 0.5 µM (dark red dashed line). (d) Toxicity testing for phenylmercuric acetate at 20 µM (red dashed line), 10 µM (green dashed line), 5 µM (blue dashed line), 1 µM (purple dashed line) and 0.5 µM (dark red dashed line).

From the 28 compounds with an average MIC below 2 µM, 17 were azoles that had already been tested in previous studies.^20^ The 11 non-azole compounds, ncs319726, IMD-0354 (IKK2 inhibitor V), broxyquinoline, clioquinol, thimerosal, broxaldine, cgp60474, tyrphostin 9, bay117085, bleomycin and phenylmercuric acetate were tested for toxicity testing in *G. mellonella* larvae. As can be seen in Figure 2, IMD-0354, broxyquinoline, clioquinol, broxaldine, cgp60474 and bay117085 were not toxic at 20 µM, since larval survival was above 80%. For ncs319726, thimerosal and tyrphostin 9, toxicity was noted at 20 µM but not at 10 µM. In contrast, bleomycin and phenylmercuric acid were toxic at concentrations as low as 0.5 µM and were disregarded for further study.

### *In vivo* efficacy of hit compounds in *M. mycetomatis-*infected *G. mellonella* larvae

**Figure 3.**
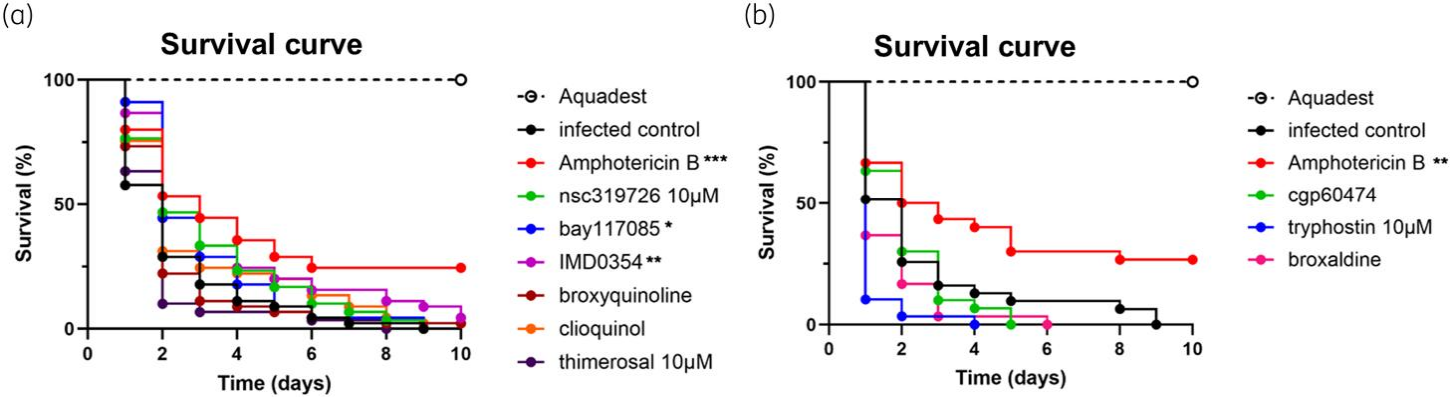
Efficacy testing of the nine selected compounds. To determine the efficacy of the selected compounds, larvae were treated either with distilled water (aquadest) (non-infected control; black dashed line), PBS (infected control; black line), amphotericin B (infected treated control; red line) or selected compound. Compared with PBS treatment, amphotericin B treatment prolonged *M. mycetomatis-*infected larvae survival within 10 days [(a) log-rank, *P* = 0.0002, ***; (b) log-rank, *P* = 0.0041, **] when injected into *G. mellonella* larvae. When compounds appeared to be toxic then concentrations were lowered to 10, 5, 1 and 0.5 µM and tested again. (a) Survival curves of *M. mycetomatis*-infected larvae treated with 10 µM ncs319726 (green line, log-rank, *P* = 0.1024), 20 µM bay117085 (blue line), 20 µM IMD-0354 (purple line), 20 µM broxyquinoline (dark red line, log-rank, *P* = 0.6994), 20 µM clioquinol (orange line, log-rank, *P* = 0.0888) and 10 µM thimerosal (dark purple line, log-rank, *P* = 0.5242). (b) Survival curves of *M. mycetomatis*-infected larvae treated with 20 µM cgp60474 (green line, log-rank, *P* = 0.7423), 10 µM tyrphostin 9 (blue line, log-rank, *P* = 0.0009) and 20 µM broxaldine (purple line, log-rank, *P* = 0.1026). Prolonged survival or reduced survival was indicated with * when the log-rank *P* value was between 0.01 and 0.05, with ** when the *P* value was between 0.001 and 0.01, and with *** when the *P* value was between 0.0001 and 0.001.

The nine non-toxic compounds, PBS and amphotericin B were tested for *in vivo* efficacy in *M. mycetomatis*-infected *G. mellonella* larvae. As expected, all *M. mycetomatis*-infected larvae treated with PBS died within 9 days of infection (Figure 3a) and amphotericin B prolonged the survival of infected larvae in Figure 3a (log-rank, *P* = 0.0002) and in Figure 3b (log-rank, *P* = 0.0041).^21^ As shown in Figure 3a, prolonged larval survival was also noted when *M. mycetomatis*-infected larvae were treated with 20 µM bay117085 (log-rank, *P* = 0.0494) or 20 µM IMD-0354 (log-rank, *P* = 0.0043). No prolonged survival was noted when *M. mycetomatis*-infected *G. mellonella* larvae were treated with 10 µM nsc319726 (log-rank, *P* = 0.1024), 20 µM broxyquinoline (log-rank, *P* = 0.6994), clioquinol (log-rank, *P* = 0.0888), thimerosal (log-rank, *P* = 0.5242) or cgp60474 (log-rank, *P* = 0.7423) or 20 µM broxaldine (log-rank, *P* = 0.1026) Figure 3a, b). Decreased survival was noted with 10 µM tyrphostin 9 (log-rank, *P* = 0.0009) (Figure 3b). Hence, although bay117085 and tyrphostin 9 are both aromatic acrylonitrile derivatives, only bay117085 showed *in vivo* antifungal activity, while tyrphostin 9 exhibited toxic effects on the host larvae.

## Discussion

**Figure 4.**
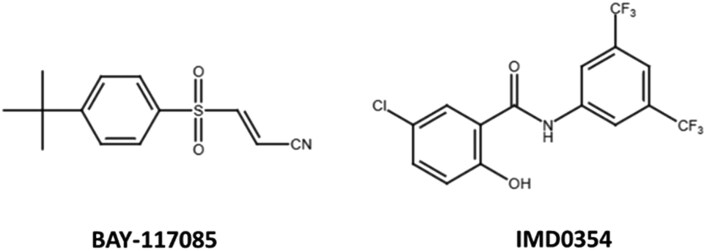
Molecular structure of bay-117085 and IMD-0354.

In this study we identified bay117085 and IMD-0354 as novel compounds that were able to prolong the survival of *M. mycetomatis*-infected larvae (Figure 4). These compounds were identified from a 5631 compound repurposing library. Due to the size of this library, we had to adopt our *in vitro* screening methodology.^19^ Previously, metabolic activity was mainly determined with XTT for *M. mycetomatis*. In this study we chose to measure luminescence with CellTiter-Glo as this can be measured directly without having to transfer the supernatant to a new plate, which is necessary when metabolic activity is measured with XTT. This lowered the workload and is less time-consuming when testing large compound libraries.^19^ Although XTT measures NADH, while luciferin measures ATP, we demonstrated before that both result in comparable results when the metabolic activity of *M. mycetomatis* is measured.^19,22–24^

Using this methodology, we demonstrated that 29 of the 5631 compounds screened inhibited the growth of *M. mycetomatis* at a concentration of 2 µM (Table 1). It was not surprising that 17 of these 29 compounds were azoles. In previous screening with the MMV compound boxes, we also identified azoles amongst the most potent compounds.^8,10,11^ The most potent azoles against *M. mycetomatis* appeared to be luliconazole (MIC_50_ of 0.001 mg/L), lanoconazole (MIC_50_ of 0.002 mg/L) and ravuconazole (MIC_50_ of 0.016 mg/L), followed by posaconazole (MIC_50_ of 0.03 mg/L) itraconazole (MIC_50_ of 0.06 mg/L) and voriconazole (MIC_50_ of 0.125 mg/L).^6,24–28^ However, despite the potent *in vitro* activity of these azoles, hardly any activity was noted when *M. mycetomatis*-infected *G. mellonella* larvae were treated with any of these azoles. No prolonged larval survival was noted when *M. mycetomatis*-infected *G. mellonella* larvae were treated with itraconazole, ketoconazole and voriconazole.^16^ Prolonged survival was noted with ravuconazole and posaconazole but only at concentrations that were higher than used in human patients.^8^ The lack of activity of itraconazole was also confirmed in a *M. mycetomatis* mouse model^21^ and in a recently published clinical trial.^5^ In this trial, no reduction in lesion size or β-glucan levels were noted during the first 6 months of treatment with either 400 mg/day itraconazole, 200 mg/week fosravuconazole or 300 mg/week fosravuconazole, only after surgery did the lesion disappear and the β-glucan levels drop.^5,29^ For azoles it is known that they do not easily penetrate fungal biofilms, therefore it is likely that the cement material in the biofilm is the reason behind this poor *in vivo* activity.^2,22^ Therefore, in this study we decided not to focus on the azoles, even though we did identify isoconazole, flutrimazole, efinaconazole, enilconazole, oxiconazole, clotrimazole and cloconazole as new azoles able to inhibit the metabolic activity of *M. mycetomatis.*

From the remaining 11 compounds with *in vitro* activity, only 2 were able to prolong larval survival. These were bay117085 and IMD-0354, which were both developed to inhibit the activation of nuclear factor κB (NF-κB) via the classical cascade. Bay117085 is an irreversible inhibitor of cytokine-inducible IκB-α phosphorylation at Ser32 and Ser36^30^ and IMD-0354 inhibits the phosphorylation of Ser177 and Ser181.^31^ NF-κB is a protein found throughout the animal kingdom and is located in the cytoplasm in its inactive dimeric form and is bound to the family of regulatory inhibitors of κB (IκB).^32^ Upon stimulation by inflammatory signals such as TNFα, the IκB kinase (IKK) complex phosphorylates the inhibitor IκB subunit. This modification marks IκB for degradation and enables nuclear translocation of the free NF-κB. Subsequently, nuclear NF-κB then binds to its target sequence and promotes transcription of target genes, such as TNF-α, MCP-1, CXCL5 and others, resulting in monocyte and neutrophil invasion into tissues.^32^ Both bay117085 and IMD-0354 thus inhibit the activation of NF-κB, and subsequently the downstream activation of inflammation.

The *in vivo* efficacy of bay117085 and IMD-0354 is therefore most likely the result of both the direct inhibition of fungal growth and the alteration of the inflammatory response in *G. mellonella* larvae. Although NF-κB is found throughout the animal kingdom, it is not present in fungi.^33,34^ Despite this, we demonstrated that both bay117085 and IMD-0354 could inhibit the growth of *M. mycetomatis in vitro*, with IC_50_ values of 1.51, 0.85 and 0.72, and 2.3, 0.4 and 1.76 µM for *M. mycetomatis* strains MM55, P1 and I1, respectively (Table 1). Also, other fungal pathogens such as *Candida albicans*, *Candida glabrata*, *Candida dubliniensis*, *Candida tropicalis*, *Candida krusei* and *Candida parapsilosis* were inhibited in growth by bay117085, with MICs ranging from 3.9 to 7.8 µM.^35^ The NF-κB inhibitor auranofin had potent antifungal activity against itraconazole-susceptible and itraconazole-resistant *Aspergillus fumigatus*, as well as inhibitory activity against *Aspergillus* biofilm formation.^36^ Both auranofin and bay117085 are able to inhibit thioredoxin reductase, which might contribute to their described antimicrobial activities.^37^ Although for IMD-0354 no antifungal activity was reported before, it is able to inhibit the growth of Gram-positive vancomycin-resistant *Staphylococcus aureus* strains with an MIC of 0.06 mg/L.^38–40^ More interestingly, IMD-0354 was also able to inhibit biofilm formation of these vancomycin-resistant staphylococci.^40^ Moreover, IMD-0354 is a salicylanilide derivative that is structurally related to the well-established anthelminthic drug niclosamide, which exhibited pronounced activities against various mycoses by disruption of fungal mitochondria function.^41^ Previously it was demonstrated that niclosamide and its new sulfanyl-substituted derivatives are active against *M. mycetomatis*.^42^ Although the activity of the recently developed sulfanyl derivatives was higher against the *M. mycetomatis* MM55 strain *in vitro*, no significantly enhanced survival was noted with any of these compounds, and thus IMD-0354 appears to be an improved salicylanilide derivative in terms of antifungal activity in *M. mycetomatis* MM55-infected *G. mellonella* larvae.

Despite the absence of any clear homologue of NF-κB, the fungal velvet proteins contain a DNA-binding domain structurally similar to NF-κB, indicating that these velvet proteins might play a similar role to NF-κB.^34^ However, the regulation of the velvet protein is most likely different from that of NF-κB, as no Iκ-B homologue has been identified in fungi yet.^34^ Therefore, it remains questionable if the velvet protein will be the targeted protein. Therefore, the fungal targets of IMD-0354 and bay117085 currently remain unknown. As mentioned before, since bay117085 and IMD-0354 will also inhibit the nuclear translocation of NF-κB in the host, the disruption of the NF-κB-regulated processes, including those in inflammation, could also have contributed to the enhanced survival. A large granulomatous inflammation zone is usually found surrounding mycetoma grains^2^ and an inflammatory reaction was also noted in the *M. mycetomatis*-infected *G. mellonella* larvae.^16^ Other fungi have been shown to induce an inflammatory response via activation of NF-κB. In human endothelial cells, the *C. albicans*-mediated chemokine expression was dependent on the NF-κB signalling pathway.^43^ The NF-κB signalling pathway was also activated when THP-1 macrophages were exposed to *A. fumigatus.*^44^ Furthermore, bay117082, a close homologue of bay117085, was able to reduce the chemotaxis, phagocytosis and killing activity of primary human neutrophils exposed to *C. albicans*.^45^ In the insect vector *Rhodnius prolixus*, IMD-0354 decreased the expression of antimicrobial peptides, resulting in a reduced mortality after *Trypanosoma cruzi* infection.^33^ This could all indicate that the increased survival of *M. mycetomatis*-infected *G. mellonella* larvae and treated with bay117085 or IMD-0354 could be the result of the direct activity of the compounds on the fungus, as well as the dampening effect on the innate immune system.

In summary, we identified bay117085 and IMD-0354 as promising therapeutic drugs for *M. mycetomatis* infections. Future studies have already been started to determine if this activity is mainly due to the inhibition of the fungal growth or the influence on the innate immune system.^46^

## Supplementary Material

dkaf369_Supplementary_Data

## Supplementary data

Table S1 is available as Supplementary data at *JAC* Online and can be found on the MycetOS github page (https://github.com/OpenSourceMycetoma/).
